# Adaptive evolution of transcription factor binding sites

**DOI:** 10.1186/1471-2148-4-42

**Published:** 2004-10-28

**Authors:** Johannes Berg, Stana Willmann, Michael Lässig

**Affiliations:** 1 Institute for Theoretical Physics, University of Cologne, 50937 Cologne, Germany

## Abstract

**Background:**

The regulation of a gene depends on the binding of transcription factors to specific sites located in the regulatory region of the gene. The generation of these binding sites and of cooperativity between them are essential building blocks in the evolution of complex regulatory networks. We study a theoretical model for the sequence evolution of binding sites by point mutations. The approach is based on biophysical models for the binding of transcription factors to DNA. Hence we derive empirically grounded fitness landscapes, which enter a population genetics model including mutations, genetic drift, and selection.

**Results:**

We show that the selection for factor binding generically leads to specific correlations between nucleotide frequencies at different positions of a binding site. We demonstrate the possibility of rapid adaptive evolution generating a new binding site for a given transcription factor by point mutations. The evolutionary time required is estimated in terms of the neutral (background) mutation rate, the selection coefficient, and the effective population size.

**Conclusions:**

The efficiency of binding site formation is seen to depend on two joint conditions: the binding site motif must be short enough and the promoter region must be long enough. These constraints on promoter architecture are indeed seen in eukaryotic systems. Furthermore, we analyse the adaptive evolution of genetic switches and of signal integration through binding cooperativity between different sites. Experimental tests of this picture involving the statistics of polymorphisms and phylogenies of sites are discussed.

## Background

The expression of a gene is controlled by other genes expressed at the same time and by external signals, a process called *gene regulation *[1]. Due to the combinatorial complexity of regulation, a large number of functional tasks can be performed by a limited number of genes. Differences in gene regulation are believed to be a major source of diversity in higher eukaryotes.

To a large extent, gene regulation is the control of transcription. It is accomplished by a number of regulatory proteins called *transcription factors *that bind to specific sites on DNA. These binding sites contain about 10 – 15 base pairs relevant for binding and are mostly located in the cis-regulatory promoter region of a gene. A cis-regulatory region in *E. coli *is about 300 base pairs long and contains a few transcription factor binding sites [2]. There may be two or more sites for the same factor in one promoter region. At the same time, the sequences of binding sites are *fuzzy*, that is, different sites for the same factor differ by about 20 – 30 percent of the bases relevant for binding [2]. This makes the identification of sites a difficult bioinformatics problem [3-5]. Frequently, the simultaneous binding at two nearby sites is energetically favoured. This so-called *binding cooperativity *can be related to various functions. In a *genetic switch *such as the famous phage lambda switch in *Escherichia coli *[6], it produces a sharp increase of the expression level at a certain threshold concentration of a transcription factor. A pair of sites for two different kinds of factors with cooperative binding can be a simple module for *signal integration*, leading to the expression of the downstream gene only when both kinds of factors are present simultaneously [1]. These examples are discussed in more detail below. Regulation in higher eukaryotes shares these features but is vastly more complicated [7]. A promoter region is typically a few thousand base pairs long and contains many different binding sites with often complex interactions. At the same time, individual sites are shorter, with about 5–8 relevant base pairs. The sites are sometimes organized in *modules *interspersed between regions containing no sites. In many known cases, the expression of a gene depends on the simultaneous presence of several factors. Well-studied examples of regulatory networks in eukaryotes include the sea urchin *Strongylocentrotus purpuratussea *[8] and the early developmental genes in *Drosophila *[9].

The sequence statistics of binding sites has been addressed in two recent theoretical studies [10,11]. Based on a model incorporating the biophysics of sequence-factor interaction [12,13], a *fitness landscape *for binding site sequences is constructed (see the discussion in the next section). The resulting mutation-selection equilibrium is analysed using a mean-field *quasispecies *approach [14]. This approach, which neglects the effects of genetic drift, is applicable in very large populations. In both studies [10,11], fuzziness is attributed to *mutational entropy *as a possible reason: the single or few sequence states with optimal binding of the transcription factor can be outweighed by the vastly higher number of sub-optimal states at some mutational distance from the optimal binding sequence. This effect is similar to the fuzziness of amino acid sequences in proteins discussed in [15].

From an evolutionary perspective, explaining the molecular programming of regulatory networks presents a striking problem. The diversification of higher eukaryotes, in particular, requires the efficient generation and alteration of regulatory binding interactions. One likely mode of evolution is gene duplications with subsequent complementary *losses of function *in both copies [16,17]. However, the differentiation of regulation should also require complementary processes that generate *new functions *of genes as a response to specific demands. This task must be accomplished mainly by sequence evolution of regulatory DNA. There are examples of highly conserved regulatory sequences with a conserved function but binding sites can also appear, disappear, or alter their sequence even between relatively closely related species; see, e.g., refs. [18-22]. This turnover of binding sites has been argued to follow an approximate molecular clock in *Drosophila *[23]. The transcription factors themselves are known to remain more conserved, especially if they are involved in the regulation of more than one gene.

The modes of regulatory sequence evolution and their relative importance remain largely to be explored. Contributions may arise from point mutations, slippage processes [24], and larger rearrangements of promoter regions [25]. The latter processes may lead to the shuffling of entire modules of binding sites between different genes. In this paper, we are more interested in the local sequence evolution within a module, which has been argued to contribute most of the promoter sequence difference between species [26]. It is also the most promising starting point for a *quantitative *analysis of binding site evolution. We study a theoretical model that takes into account point mutations, selection, and genetic drift. The form of selection is inferred from the biophysics of the binding interactions between transcription factors and DNA. We derive the stationary distribution of binding sites under selection, which shows specific correlations between nucleotide frequencies at different positions in a binding site. The non-stationary solutions of the model describe efficient adaptive pathways for the molecular evolution of regulatory networks by point mutations. This efficiency can be quantified in terms of the length of the binding motif, and the length of the promoter region, and the fitness landscape for factor binding, which is amenable to quite explicit modeling.

With the parameters found in natural systems, our model predicts that a new binding site for a given transcription factor can be generated by a fast series of adaptive substitutions, even if the expression of the corresponding gene bears even a modest fitness advantage. The evolutionary time required for site formation in response to a *newly arising *selection pressure is estimated in terms of the characteristic time scales of mutation, selection, and drift. For *Drosophila*, it may be as short as 10^5 ^years even for moderate selection pressures. However, this pathway is found to depend crucially on the presence of selection. It would be too slow under neutral evolution, in contrast to the results of [7], see also the recent discussion in [27]. Cooperative interactions between binding sites can evolve adaptively on similar time scales, as we show for the two simple examples alluded to above, the genetic switch and the signal integration module. These results are discussed at the end of the paper with particular emphasis on possible experimental tests.

### Factor binding and selection

The binding energy (measured in units of *k*_*B*_*T*) between a transcription factor and its binding site is, to a good approximation, the sum of independent contributions from a small number of important positions of the binding site sequence, , with  ≈ 10 - 15 [28-30]. The individual contributions *ε*_*i *_depend on the position *i *and on the nucleotide *a*_*i *_at that position. There is typically one particular nucleotide  preferred for binding; the sequence  is called the *target sequence*. The target sequence can be inferred as the consensus sequence of a sufficiently large number of equivalent sites. The so-called *energy matrix ε*_*i*_(*a*) has been determined experimentally for some factors from *in vitro *measurements of the binding affinity for each single-nucleotide mutant of the target sequence. Typical values for the loss in binding energy are 1–3 *k*_*B*_*T *per single-nucleotide mismatch away from the target sequence. In this paper, we use the further approximation *ε*_*i *_= *ε *if *a*_*i *_=  and *ε *= 0 otherwise, the so-called *two-state model *[12]. The binding energy of any sequence  is then, up to an irrelevant constant, simply given by its Hamming distance *r *to the target sequence: *E*/*k*_*B*_*T *= *εr*. (The Hamming distance is defined as the number of positions with a mismatch *a*_*i *_≠ .)

It is important to note the status of this "minimal model" of binding energies for the discussion in this paper. Both approximations underlying the model can be violated. Even though typical mismatch energies are of the same order of magnitude, there can be considerable differences between different substitutions at one position and between different nucleotide positions. Moreover, deviations from the approximate additivity of binding energies for the single nucleotide positions have also been observed. However, these complications do not affect the order-of-magnitude estimates for adaptive sequence evolution. As it will become clear, the efficiency of binding site formation depends only on the qualitative shape of the fitness landscapes derived below. In these landscapes, the regime of weakly-binding sequences and of strongly-binding sequences are separated by only a few single nucleotide substitutions. The relative magnitude of the fitness increase of these substitutions does not matter in first approximation. Indeed, inhomogeneities in the values of the *ε*_*i*_(*a*) tend to reduce the number of *crucial *steps in the adaptive process and thereby to further increase its speed.

Within the two-state model, the binding probability of the factor in thermodynamic equilibrium is



Here *ε *is the binding energy per nucleotide mismatch and *ερ *is the chemical potential measuring the factor concentration. Both parameters are expressed in units of *k*_*B*_*T *and hence dimensionless. Appropriate values for typical binding sites have been discussed extensively in refs. [10,13]. It is found that *ε *should take values around 2, which is consistent with the measurements for known transcription factors mentioned above [28-30]. The chemical potential depends on the number of transcription factors present in the cell, on the binding probability to *background *sites elsewhere in the genome (which have a sequence similar to the target sequence by chance), and on the *functional *sites in the in the genome other than the binding site in question that may compete for the same protein. Binding to background sites does not significantly reduce the binding to a specific functional site [13]. This leads to values *ρ *≈ (log *n*_*f*_)/*ε *≈ 2 - 4, given observed factor numbers *n*_*f *_of about 50 – 5000 [13]. Binding to other copies of the same functional sequence becomes only relevant at low factor concentrations and high number of copies, when sites compete for factors.

A *fitness landscape *quantifies the fitness *F* of each sequence state at the binding site. Fitness differences arise due to different expression levels of the regulated gene, and these in turn depend on the binding of the transcription factors. It is only these fitness differences that enter the population dynamics   of binding site sequences in the next section. Following the conceptual framework of ref. [10], we assume that the environment of the regulated gene can be described by a number of *cellular states *(labelled by the index *α*) with different transcription factor concentrations, i.e., with different chemical potentials *ρ*^*α*^. These cellular states can be thought of as different stages within a cell cycle. In each state, the fitness depends on the expression level of the regulated gene in a specific way. This expression level is determined by the binding probability *p*^*α *^of the transcription factor. Assuming that both dependencies are linear (this is not crucial) and that the cellular states contribute additively to the overall fitness *F*, we obtain



Here the *selection coefficient s*^*α *^is defined as the fitness difference (due to different expression of the downstream gene) between the cases of complete factor binding and no binding in the state *α*. Such fitness differences can now be measured directly in viral systems [31]. Inserting (1), the fitness becomes a function of the Hamming distance *r *only. We note that the fitness *F *is measured relative to that of a phenotype with zero binding probability in any state *α*.

**Figure 1:**
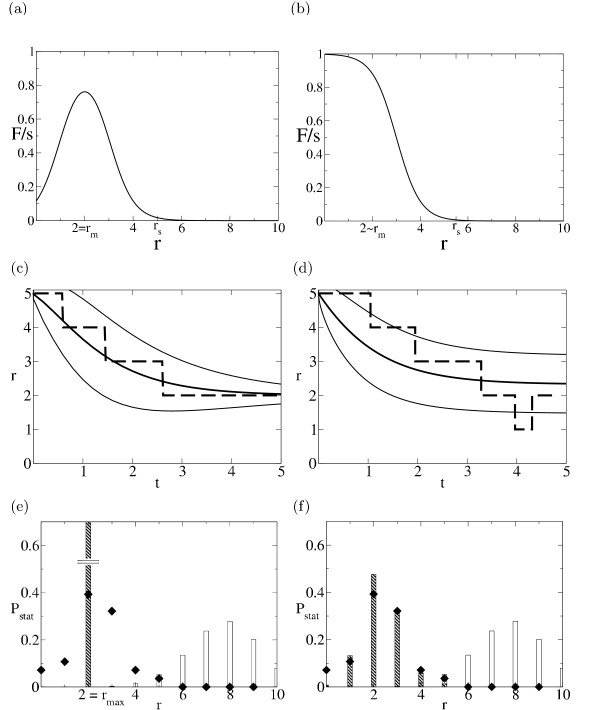
**Fitness landscapes and adaptive evolution for a single binding site **(a) *Crater *landscape (3) and (b) *Mesa *landscape (4), as a function of the Hamming distance *r *from the target sequence (within the approximation of the two-state model). *r*_m _gives the point where the binding probability reaches a maximum (crater landscape), or else values close to 1 (mesa landscape). *r*_*s *_approximately indicates the onset of selection, i.e. a binding probability appreciably different from zero. (c) Adaptive dynamics as a function of time *t *measured in units of 1/(2*sμN*) in the crater landscape at strong selection (*sN *= 100). Single history *r*(*t*) (dashed lines), ensemble average  (thick solid lines) and width given by the standard deviation curves  ± *δr*(*t*) (thin solid lines), (d) Same as (c) in the mesa landscape at moderate (*sN *= 6.8) selection, (e) Stationary ensembles *P*_stat_(*r*) of binding site sequences with in the crater landscape at strong selection (filled bars) and for neutral evolution (empty bars). (f) Same as (e) in the mesa landscape at moderate selection, together with the histogram of Hamming distances of CRP site sequences in *E. coli *from their consensus sequence (diamonds, from [10]).

In a simple case, there are just two relevant cellular states. The *on *state favours expression of the gene, the *off *state disfavours it. It is then natural to assume selection coefficients of similar magnitude; here we take for simplicity *s *= *s*^on ^= -*s*^off ^> 0. We then obtain a *crater *landscape,



with a high-fitness rim between *ρ*^off ^and *ρ*^on ^flanked by two sigmoid thresholds; see fig. 1(a). The generic features of this fitness landscape are easy to interpret: the two-state selection assumed here favors intermediate binding strength (i.e., intermediate Hamming distances *r*) where binding occurs and the gene is expressed in the *on state *but not in the *off *state. Sequences with large Hamming distance *r *>*ρ*_on _can bind the factor neither in the *on *nor in the *off *state, while sequences with *r *<*ρ*_off _lead to binding in the *on *and the *off *state. Both cases lead to misregulation of the downstream gene, and hence to a lower fitness. We note that the key feature of these fitness landscapes, the sigmoid thresholds, is independent of the particular choices of *s*^on ^and *s*^off^.

An even simpler fitness landscape is obtained if only the *on *state contributes significantly to selection, i.e., if *s *= *s*^on ^> 0 and *s*^off ^= 0. The crater landscape then reduces to the *mesa *landscape discussed in [10,32],



which has a high-fitness plateau of radius *ρ *and one sigmoid threshold; see fig. 1(b). In this case, all sequences with sufficiently small Hamming distance to the target sequence (*r *<*ρ*^on^) have a high fitness. In both cases, the parameters of the binding model have a simple geometric interpretation: *ε *gives the slope and the *ρ*^*α *^give the positions of the sigmoid thresholds in the fitness landscape. Eqs. (3) and (4) are again to be understood as minimal models of fitness landscapes for binding sites, representing target sequence selection for a given level of binding (*ρ*^off ^<*r *<*ρ*^on^) and for sufficiently strong binding (*r *<*ρ*^on^), respectively. Despite its simplicity, this type of selection model based on biophysical binding affinities is nontrivial from a population-genetic viewpoint since it leads to generic correlations between frequencies of nucleotides *a*_*i *_and *a*_*j *_within a site, see the Results section below. We will also study generalized models with correlations between two sites generated by cooperative binding. On the other hand, these models neglect the context dependence of the binding process through cofactors and chromatin structure. However, they are a good starting point for order-of magnitude estimates of the adaptive evolution of binding sites.

### Mutation, selection, and genetic drift

The rates of nucleotide point mutation show a great variation, ranging from *μ *~ 10^-4 ^per site and generation for RNA viruses to values several orders of magnitude lower in eukaryotes, e.g. *μ *≈ 2 × 10^-9 ^in *Drosophila *[33]. (Here we model mutation as a single-parameter Markov process; we do not distinguish between transitions and transversions.) The evolution of a sufficiently large population under mutation and selection can be described in terms of the average fraction of the population with a given binding sequence. This so-called mean-field approach neglects the fluctuations due to finite population size (genetic drift). It leads to the so-called *quasispecies *theory [14]. For a population of sequences at a single binding site, the quasispecies population equation can be written for the fraction *n*(*r*,*t*) of individuals at Hamming distance *r *from the target sequence at time *t*. Along with a generalisation for two binding sites, it has been analysed in detail in ref. [10]. For the mesa landscape, the stationary solution *n*_stat_(*r*) has been found exactly [32]. It depends only on the ratio *s*/*μ *and describes a stable *polymorphic *population, i.e., several sequence states coexist. The mean-field approach is valid as long as the stochastic reproductive fluctuations are leveled out by mutations. This requires absolute population numbers *Nn*_stat_(*r*) ≫ 1/*μ *for all relevant *r*, a stringent condition on the total population size *N*.

This paper is concerned with a different regime of population dynamics, as described by the Kimura-Ohta theory for finite populations evolving by stochastic fluctuations (genetic drift) and selection [34-36]. According to this theory, a new mutant with a fitness difference Δ*F *relative to the pre-existing allele could spread to fixation in the population. This is a stochastic process, whose rate constant is given by



in a diffusion approximation valid for Δ*F *≪ 1 [37]. Here *N *is the *effective *population size (with an additional factor 2 for diploid populations). Eq. (5) has three well-known regimes. For substantially *deleterious *mutations (*N*Δ*F *≲ - 1), substitutions are exponentially suppressed. *Nearly neutral *substitutions (*N*|Δ*F*| ≪ 1) occur at a rate *u *≈ *μ *approximately equal to the rate of mutations in an individual. For substantially *beneficial *mutations (*N*Δ*F *≳ 1), the substitution rate is enhanced, with *u *≃ 2*μN*Δ*F *for *N*Δ*F *≫ 1.

In this picture, a population has a monomorphic majority for most of the time and occasional coexistence of two sequence states while a substitution is going on. The time of coexistence is *T *~ *N *for nearly neutral and *T *~ 1/Δ*F *for strongly beneficial substitutions. The picture is thus self-consistent for *Tu *≪ 1, i.e., for *μN *≪ 1. Asymptotically, it describes monomorphic populations moving through sequence space with hopping rates *u*.

Introducing an *ensemble *of independent populations, this stochastic evolution takes the form of a Master equation. For a single binding site, we obtain



Here *P*(*r*,*t*) denotes the probability of finding a population at Hamming distance *r *from the target sequence, and *u*_*r*,*r' *_is given by (5) with Δ*F *= *F*(*r'*) - *F*(*r*). The combinatorial coefficients arise since a sequence at Hamming distance *r *can mutate in  different ways that increase *r*, and in *r *ways that decrease *r*, where *c *= 4 is the number of different nucleotides. The stationary distribution is

*P*_stat_(*r*) ~ exp[*S*(*r*) + 2*NF*(*r*)].     (7)

Here  is the *mutational entropy *(the log fraction of sequence states with Hamming distance *r*) [32] and we have used the exact result *u*_*r*+1,*r*_/*u*_*r*,*r*+1 _= *e*^2(*N *- 1)Δ*F*^. To derive (7), we then simply approximated *N *- 1 by *N*. The form of *P*_stat_(*r*) reflects the selection pressure, i.e., the scale *s *of fitness differences in the landscape *F*(*r*). For neutral evolution (2*sN *= 0), the stationary distribution



is obtained from a flat distribution over all sequence states. For moderate selection (2*sN *~ 1), *P*_stat_(*r*) results from a nontrivial balance of stochasticity and selection. For strong selection (2*sN *≫ 1), *P*_stat_(*r*) takes appreciable values only at points of near-maximal fitness, where *F*(*r*) ≳ *F*_m _- 1/2*sN*. In this regime, the dynamics of a population consists of beneficial mutations only, i.e., the system moves uphill on its fitness landscape.

The Master equation (6) and the mean-field quasispecies equation thus describe opposite asymptotic regimes, *μN *≪ 1 and *μN *≫ 1, of the evolutionary dynamics. Effective population sizes show a large variation, from values of order 10^9 ^in viral systems to *N *~ 10^6 ^in *Drosophila *and *N *~ 10^4 ^- 10^5 ^in vertebrates. (These numbers bear some uncertainty; one reason is that TV varies across the genome [38].) We conclude that the mean-field quasispecies is well suited for viral systems, while eukaryotes clearly show a stochastic dynamics of substitutions.

## Results and discussion

### Stationary distributions and nucleotide frequency correlations

In the previous sections, we have expressed the fitness landscape and the resulting population distributions as a function of the Hamming distance *r *because it is a convenient parameterization of the binding energy in the two-state model. In order to compare this approach to standard population genetics, it is useful to recast eq. (7) for the elementary sequence states (*a*_1_,...,*a*_*l*_),



where the sum runs over all sequence states at fixed *r*. At neutrality, the distribution over sequence states factorizes in the single nucleotide positions,



In the specific case of the two-state model, *ν*_0_(*a*_*i*_) is simply a flat distribution over nucleotides but it is obvious how this form can be generalized to arbitrary nucleotide frequencies.

According to eq. (7), the stationary distribution under selection takes the form



The salient point is that *F*(*r*) is generically a strongly nonlinear function of *r *due to the sigmoid dependence of the binding probability on *r*. An analogous statement holds beyond the two-state approximation for the dependence of *F *on the binding energy *E*. Hence, even if (*a*_1_,...,*a*_*l*_) factorizes in the single nucleotide positions, (*a*_1_,...,*a*_*l*_) does not. The selection introduces specific correlations between the nucleotides: the fitness differences and, hence, the nucleotide frequencies at one position *i *depend on all other it *l *- 1 positions in the motif.

### Adaptive generation of a binding site

We now apply the dynamics (6) to the problem of adaptively generating a binding site in response to a newly arising selection pressure. We study a case of strong selection (*sN *= 100) in the crater fitness landscape (3) with parameters  = 10, *ε *= 2, *ρ*^on ^= 3, *ρ*^off ^= 1 (implying that the factor concentrations differ by a factor of 50), and a case of moderate selection (*sN *= 7) in the mesa landscape with parameters  = 10, *ε *= 1, *ρ *= 3.6. (The mesa type may be most appropriate for factors with multiple binding sites such as the CRP repressor in *E. coli*, where binding to an individual site is negligible in the *off *state.) The fitness landscapes for both cases are shown in fig. 1(a),1(b) in units of the selection pressure *s*. Substantially beneficial mutations occur only on their sigmoid slopes, i.e., in narrow ranges of *r*. The upper boundary of this region is given by *r*_*s *_= *ρ*^on ^+ log[*sN*(*e*^*ε *^- 1)]/*ε *which takes typical values *r*_*s *_= 5 - 7. In fig. 1(c),1(d), we show a sample history of adaptive substitutions from *r *= 5 to lower values of r, which are close to the point *r*_m _of maximal fitness. The statistics of this adaptation is governed by the ensemble *P*(*r*, *t*); the average  and the standard deviation *δr*(t) appear also in fig. 1(c),1(d). In the case of strong selection, the expected time of the adaptive process is readily estimated in terms of the uphill rates in (6),



and takes values of a few times 1/*sμN*. We emphasize again that this simple form depends only on the qualitative form of the fitness landscape, namely, that weakly and strongly binding sequence states are separated only by few point mutations. The conclusions are thus largely independent of the details of the fitness landscape, which justifies using the two-state approximation.

Can such a selective process actually happen? This depends on the initial state of the promoter region in question *before *the selection pressure for a new site sets in. The region is approximated as an ensemble of *L*_1 _= *L *-  + 1 candidate sites undergoing *independent *neutral evolution, i.e., the simultaneous updating of  sites by one mutation is replaced by independent mutations. The length of the promoter region is denoted by *L*. At stationarity, the Hamming distance at a random site then follows the distribution *P*_stat_(*r*) ~ exp[*S*(*r*)] shown as empty bars in fig. 1(e),1(f). The minimal distance *r*_min _in the entire region is given by the distribution , where  is the cumulative distribution for a single site.  is found to be strongly peaked, taking appreciable values only in the range  around its average. We assume selective evolution sets in as soon as at least one site has a Hamming distance *r *≤ *r*_*s*_. This is likely to happen spontaneously if , leading to a joint condition on , *L*, and *r*_*s*_. For , there is a neutral waiting time before the onset of adaptation. Its expectation value



is calculated in the appendix. It is generically much larger than the adaptation time *T*_*s*_, rendering the effective generation of a new site less feasible.

The stationary distribution *P*_stat_(*r*) under selection is given by (7) and shown as filled bars in fig. 1(e),1(f). For strong selection, it is peaked at the point *r*_m _of maximal fitness. For moderate selection, it takes appreciable values for *r *= 0 - 4: the binding site sequences are *fuzzy*. Assuming that the CRP sites at different positions in the genome of *E. coli *have to a certain extent evolved independently, we can fit *P*_stat_(*r*) with their distance distribution (data taken from [10]). At the values of *ε *and *ρ*^on ^chosen, the two distributions fit well, see fig. 1(f). This finding is discussed in more detail below.

### Adaptation of binding cooperativity

The cooperative binding of transcription factors involves protein-protein interactions which may be specific to the DNA substrate. These interactions often do not require conformational changes of either protein involved and depend only on few specific contact points. They result in a modest energy gain of order 3 – 4*k*_*B*_*T *[1]. Hence, it is a reasonable simplification to study the adaptive adjustment of binding affinities using a simple generalisation of the two-state binding model. We define the energies *E*_1_/*k*_*B*_*T *= *εr*_1 _and *E*_2_/*k*_*B*_*T *= *εr*_2 _for the binding of a single factor and  for the simultaneous binding of both factors. The cooperativity gain is assumed to result from mutations at  positions in the DNA sequences of the factors, which encode the amino acids at the protein-protein contact points. These mutations define a Hamming distance  from the target sequence for optimal protein-protein binding, and 2*γε*/ is the binding energy per nucleotide. Here we use the values *ε *= 2,  = 6 and *γ *= 1 but the qualitative patterns shown below are rather robust.

The resulting equilibrium probabilities for the four thermodynamic states (--) (both factors unbound), (+-) and (-+) (one factor bound), and (++) (both factors bound) are

*q--*,

*q*_+- _= *q*_-- _exp[-*ε*(*r*_1 _- *ρ*_1_)],

*q*_+- _= *q*_-- _exp[-*ε*(*r*_2 _- *ρ*_2_)],     (14)

*q*_++ _= *q*_-- _exp[-*ε*(*r*_1 _+ *r*_2 _- *ρ*_1 _- *ρ*_2 _- 2*γ*)],

with the normalisation *q*_-- _+ *q*_+- _+ *q*_-+ _+ *q*_++ _= 1. The scaled chemical potentials *ρ*_1 _and *ρ*_2 _are independent variables if the two sites bind to different kinds of factors and are equal if they bind to the same kind. As before, the binding probabilities determine expression levels and, therefore, the fitness. Here we study only pairs of sites contributing additively to the expression level in each cellular state, where we have



Other important cases include activator-repressor site pairs such as the famous *lac *operon [39], where the transcription-factor induced expression level is proportional to *q*_+-_. The stochastic dynamics of substitutions is straightforward to generalise; it leads to a Master equation like (6) for the joint distribution *P*(*r*_1_, *r*_2_, , *t*). This higher-dimensional equation can again be solved exactly for its steady state

*P*_stat_(*r*_1_, *r*_2_, ) ~ exp[*S*(*r*_1_) + *S*(*r*_2_) + *S*() + 2*NF*(*r*_1_, *r*_2_, )].     (16)

**Figure 2:**
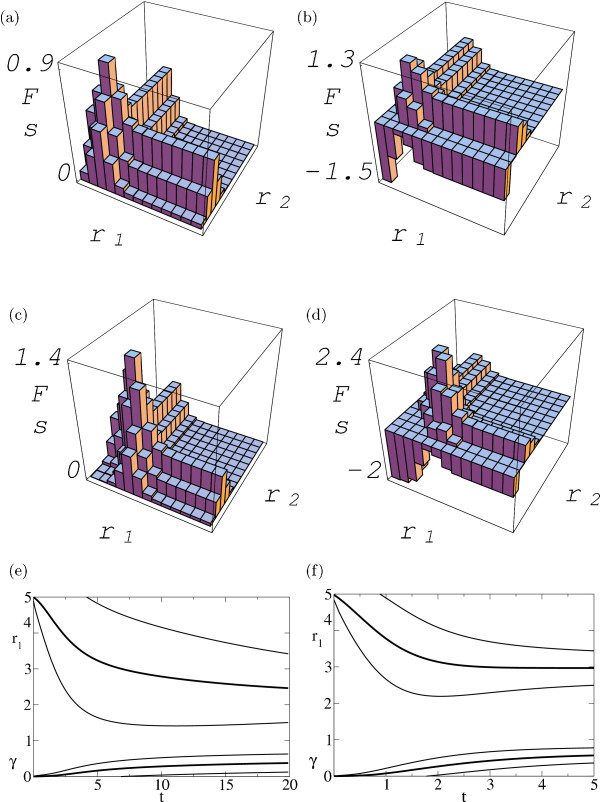
**Fitness landscapes and adaptive evolution for a pair of sites with cooperative binding. **Genetic switch (left column), signal integration module (right column). (a,b) Fitness landscape *F*(*r*_1_, *r*_2_) without cooperativity (*γ *= 0). (c,d) Fitness landscape. *F*(*r*_1_, *r*_2_) with cooperativity (*γ *= 1). Next-nearest neighbour states (*r*_1_, *r*_2_) and  of similar fitness are linked by *compensatory *mutations if the intermediate states (*r*_1_, ) and (, *r*_2_) have lower fitness. (e,f) Adaptive dynamics: ensemble averages  and  (thick lines), ensemble width given by  (same for *r*_2 _and  (thin lines); cf. fig. 1(e,f).

Here we discuss two simple examples of fitness landscapes where binding cooperativity evolves by adaptation to specific functional demands. A *genetic switch *with a sharp expression threshold is favoured in a system with a single transcription factor having similar concentrations in its *on *and *off *cellular state. As can be seen from eq. (14), cooperative binding can sharpen the response of the binding probability to variations in factor concentration, *q*_++ _~ 1/[1 + exp(-2*ερ *+ ...)] versus *p *~ 1/[1 + exp(-*ερ *+ ...)] as given by (1) for individual binding. Figs. 2(a),2(c) show the fitness landscape *F*(*r*_1_, *r*_2_, *γ*) obtained from (14) and (15) for *ρ*^on ^= 2.5, *ρ*^off ^= 1.5, and *s *= *s*^on ^= -*s*^off^. A simple *signal integration module *responds to two different factors in four different cellular states, (*on*, *on*), (*on*, *off*), (*off*, *on*), (*off*, *off*). Individually weak but cooperative binding leads to expression of the gene only if both factors are present simultaneously. This case is favoured by a fitness function of the form (15) with selection coefficients *s *= -*s*^off,off ^= -s^on,off ^= -s^off,on ^= *s*^on,on^/2. The resulting fitness landscape *F*(*r*_1_, *r*_2_, *γ*) is shown in figs. 2(b),2(d) for chemical potentials *ρ*^on ^= 3, *ρ*^off ^= 1 (for each factor).

In both cases, a pair of sites with weaker individual binding (*r*_1_, *r*_2 _= 3 - 4) and cooperativity (*γ *= 1) is seen to have a higher fitness than an optimal pair (*r*_1 _= *r*_2 _= 2) without cooperativity, as expected. Adaptive pathways  and  for strong selection (*sN *= 100) are shown in fig. 2(e),2(f). Typical adaptation times *T*_*s *_are again a few times 1/(*sμN*). A closer look reveals that this fast adaptation sometimes leads to a *metastable *local fitness maximum with some degree of cooperativity. *Compensatory *mutations (see below) are then required to reach the global maximum, a process that may be considerably slower. The fuzziness *δr*_1,2_(*t*) and *δγ*(*t*) observed in fig. 2(e),2(f) decays on the larger time scale of compensatory mutations, reflecting the presence of such metastable states.

## Conclusions

Transcription factors and their binding sites emerge as a suitable starting point for quantitative studies of gene regulation. Binding site sequences are short and their sequence space is simple. Moreover, the link between sequence, binding affinity, and fitness is experimentally accessible. For a single site, the simplest examples are of the *mesa *[10] or of the *crater *type, see fig. 1(a),1(b). Landscapes for a pair of sites with cooperative binding interactions are of a similar kind as shown in fig. 2(a),2(b),2(c),2(d). They can be used to predict the outcome of specific single-site mutation experiments to a certain extent.

### Fast adaptation may generate or eliminate a new binding site

Despite this simplicity, the evolutionary dynamics of binding sites is far from trivial, since it is governed, in the generic case, by the interplay of three evolutionary forces: selection, mutation, and genetic drift. Here we have focused on the dynamical regime appropriate for eukaryotes, where the evolution can be approximated as a stochastic process of substitutions. We find the possibility of selective pathways generating a new site in response to a newly arising selection pressure, starting from a neutrally evolved initial state and progressing by point substitutions. Such a selective formation takes roughly *T*_*s *_≈ Δ*r*/(2*sμN*) generations, where Δ*r *is the number of adaptive substitutions required. This number is given by the Hamming distance between the onset of selection and the point of optimal fitness, Δ*r *= *r*_*s *_- *r*_*m*_, and takes values 2 – 3 for typical fitness landscapes; see fig. 1(a),1(b). For *Drosophila melanogaster*, with *μ *≈ 2 × 10^-9 ^[33] and *N *≈ 10^6^, the resulting *T*_*s *_is of the order of 10^6 ^generations or 10^5 ^years even for sites with a relatively small selection coefficient *s *= 10^-3^. Such selective processes are faster than neutral evolution by a factor of about 1000 and would allow for independent generation of sites even after the split from its closest relative *Drosophila simulans *about 2.5 × 10^6 ^years ago. Notice that new sites are more readily generated in large populations. As discussed above, generating a new site may also require a neutral waiting time to *T*_0 _until at least one candidate site in the promoter region of the gene in question reaches the threshold distance *r*_*s *_from the target sequence, where selection sets in. For site formation to be efficient, however, selection must be able to set in spontaneously, i.e., *T*_0 _must not greatly exceed the adaptive time *T*_*s*_. This places a bound on the relevant length  of the binding motif that can readily form in a promoter region of length *L*. Given *L *≈ 300, for example, a motif with  = 8 and *r*_*s *_= 3 could still allow for spontaneous adaptive site formation. (For longer motifs, corresponding to groups of sites with fixed relative distance, this pathway would require promoter regions of much larger *L*.) A more general case has recently been treated numerically in [27], where the dependence of the neutral waiting time on the *G/C *ratio of the initial sequence has been investigated. One may speculate that this adaptive dynamics is indeed one of the factors influencing the length of regulatory modules in higher eukaryotes. Clearly, the present model also allows for pathways of *negative selection *leading to the elimination of spurious binding sites in regulatory or non-regulatory DNA where the binding has an adverse fitness effect. This is important since under neutral evolution, candidate sites with a distance of at most *r*_*s *_from the target sequence occur frequently on a genome-wide scale. A recent study has indeed found evidence for such negative selection from the underrepresentation of binding site motifs over the entire genome [40].

### Binding sites under selection have nucleotide frequency correlations

We have shown that under stationary selection the frequencies of nucleotides at any two positions of the binding sequence are correlated. For the two-state model, the correlations are the same for any pair of positions *i *≠ *j *and can be computed exactly from the joint distribution (11). We emphasize that these correlations refer to an ensemble of independently evolving (monomorphic) populations and are not to be confused with linkage disequilibria within one population. This finding limits the accuracy of bioinformatic weight matrices, which are often assumed to factorize in the nucleotide positions even in the presence of selection.

### Experimental tests: binding site polymorphisms and phylogenies

The predictions of our model lend themselves to a number of experimental tests. In the dynamical regime appropriate for eukaryotes (*μN *≪ 1), populations should be monomorphic at most positions of their binding site sequences and polymorphic at a few. On the other hand, the quasispecies model discussed in refs. [10,11] (which assumes *μN *≫ 1) may be most appropriate in viral systems. The intermediate regime *μN *~ 1 with frequent polymorphisms *and *genetic drift could be realized in some bacterial systems and presents a challenge for theory. Thus it would be very interesting to compare the statistics of single-nucleotide polymorphisms at binding sites in eukaryotes, bacteria, and viruses. Polymorphism data are expected to contain evidence for adaptive evolution. However, statistical tests of selection must be modified for promoter sequences [40,41]. A recent study uses data on binding sites in three yeast species and deduces the rates of sequence evolution [42].

A complementary source of information are phylogenies of binding sites. Trees with functional differences between branches contain information on the generation of new sites or of interactions between sites and on the time scales involved. In a tree for a conserved site or group of sites with sufficiently long branches, the fuzziness of the sequences observed on different branches is given by the ensemble *P*_stat _introduced above. For strong selection, *P*_stat _lives on the *quasi-neutral *network of sequence states with maximal fitness, where two neighbouring sequence states are linked by neutral mutations or by pairs of *compensatory *mutations at two different positions. In the crater landscape for a single site, this quasi-neutral network consists of all sequences with a fixed distance *r *= *r*_max _from the target sequence; see fig. 1(a). Beyond the two-state approximation for binding energies, it will be smaller since only some of the positions are energetically equivalent. For a group of sites, however, quasi-neutral networks can be larger since compensatory mutations can also take place at positions on different sites as shown in fig. 2(d) for the example of a signal integration module. This is consistent with experimental evidence that the sequence divergence between *Drosophila melanogaster *and *Drosophila pseudoobscura *involves compensatory mutations and stabilising selection between different binding sites [43].

For weaker selection, site fuzziness increases further since *P*_stat _extends beyond the sequence states of maximal fitness and is influenced by mutational entropy. As shown in fig. 1(f), one can explain in this way the observed fuzziness in CRP sites of *E. coli*. It would then reflect different evolutionary histories of independent populations, rather than sampling in one polymorphic population as in the quasispecies picture of refs. [10,11]. (In a mean-field quasispecies, appreciable fuzziness occurs only for selection coefficients *s *~ *μ*, minute in other than viral systems.) However, the data are also compatible with strong selection if the selection coefficients *s*^*α*^, and hence the value of *r*_m_, vary between different genes. Clearly, comparing *P*_stat _with the distribution of sites in a single genome requires the assumption that the evolutionary histories of sites at different positions are at least to some extent independent. Future data of orthologous sites in a sufficient number of species will be more informative. Thus, further experimental evidence is needed to clarify the role of mutational entropy in the observed fuzziness.

### Evolvability of binding sites

The present work was aimed at obtaining some insight into the molecular mechanisms and constraints underlying the dynamics of complex regulatory networks, thereby quantifying the notion of their *evolvability*. The programming of binding sites and of cooperative interactions between them is found to provide efficient modes of adaptive evolution whose tempo can be quantified for the case of point mutations. The formation of complicated signal integration patterns and of multi-factor interactions in higher eukaryotes, however, requires generalizing our arguments in two ways. There are further modes of sequence evolution such as slippage events, insertions and deletions, large scale relocation of promoter regions, and recombination. Our ongoing work is aimed at quantifying their relative importance in terms of substitution rates. Moreover, there are also more general fitness landscapes describing, e.g., binding sites interacting via the expression level of the regulated gene (such as activator-repressor site pairs) and the coupled evolution of binding sites in different genes.

The rapid evolution of networks hinges upon the existence of adaptive pathways for these formative steps with a characteristic time scale *T*_*s *_~ 1/(*sμN*) much smaller than *T*_0 _~ 1/*μ*, the time scale of neutral evolution. The presence of these two time scales has a further interesting consequence. If the selection pressure on an existing site ceases, that site will disappear on the larger time scale *T*_0_. It is possible, therefore, that large existing networks have accumulated a considerable number of *redundant *regulatory interactions acquired by selection in their past. This may be one factor contributing to their robustness against perturbations.

## Methods – neutral evolution of binding sites

To estimate the average neutral waiting time *T*_0_, we study the mutation dynamics in the restricted range *r *= *r*_*s *_+ 1,...,, allowing mutations from *r*_*s *_+ 1 to *r*_*s *_but suppressing mutations from *r*_*s *_back to *r*_*s *_+ 1. We evaluate the time-dependent solution *P*(*r*, *t*) of the Master equation (6) with the initial condition *P*(*r*, 0) = *P*_stat_(*r*), and the resulting cumulative probability . The current across the lower boundary, *J*(*t*) = *μ*(*r*_*s *_+ 1)*P*(*r*_*s *_+ 1,*t*) = -*dQ*/*dt*, determines the waiting time for a single site,



This is formally solved by expanding in eigenfunctions of the mutation operator. In the case relevant here, the system remains close to equilibrium since the boundary current is much smaller than typical currents for *r *≥ *r*_*s*_. Hence, *P*(*r*,*t*) ≈ *P*_stat_(*r*) exp(-*λt*) with *λ *= *J*(0)/*Q*(0) = *μ*(*r*_*s *_+ 1)*P*_stat_(*r*_*s *_+ 1)/*Q*_stat_(*r*_*s *_+ 1). We conclude that the waiting time for a single site is positive with probability *Q*_stat_(*r*_*s *_+ 1), following a distribution ~exp(-*λt*), and 0 otherwise. The resulting expectation value is *T*_0 _= *Q*_stat_(*r*_*s *_+ 1)/*λ*. For *L*_1 _independent sites, the distribution of positive waiting times is still exponential, and to is given by an expression of the form (17) with a total boundary current . This yields  as given by (13). The average waiting time (in units of 1/*μ*) becomes large for values of *r*_*s *_in the tail of the distribution , where . This is the case for .

## Authors' contributions

JB carried out analytical and numerical work, SW performed numerical work and data processing. ML conceived of the study, carried out analytical work, and coordinated the project. All authors read and approved the final manuscript.

## References

[B1] Ptashne M, Gann A (2002). Genes and Signals.

[B2] Collado-Vides J, Magasanik B, Gralla J (1991). Control site location and transcriptional regulation in Escherichia coli. Microbiol Reviews.

[B3] Bussemaker H, Li H, Siggia ED (2000). Building a dictionary for genomes: Identification of presumptive regulatory sites by statistical analysis. Proc Nat Acad Sci USA.

[B4] Hertz G, Storrno G (1999). Identifying DNA and protein patterns with statistically significant alignments of multiple sequences. Bioinformatics.

[B5] Stormo GD, Fields D (1998). Specificity, energy and information in DNA-protein interactions. Trends Biochem Sci.

[B6] Ptashne M (1992). A genetic switch: Phage λ and higher organisms.

[B7] Stone J, Wray G (2001). Rapid Evolution of cis-Regulatory Sequences via Local Point Mutations. Mol Biol Evol.

[B8] Davidson E (1999). A view from the genome :Spatial control of transcription in sea urchin development. Current Opinion in Genetics & Development.

[B9] Tautz D (2000). Evolution of transcriptional regulation. Current Opinion in Genetics & Development.

[B10] Gerland U, Hwa T (2002). On the Selection and Evolution of Regulatory DNA Motifs. J Mol Evol.

[B11] Sengupta A, Djordjevic M, Shraiman B (2002). Specificity and robustness in transcription control networks. Proc Nat Acad Sci USA.

[B12] Berg O, von Hippel P (1987). Selection of DNA binding sites by regulatory proteins. J Mol Biol.

[B13] Gerland U, Moroz D, Hwa T (2002). Physical constraints and functional characteristics of transcription factor-DNA interaction. Proc Nat Acad Sci USA.

[B14] Eigen M, McCaskill J, Schuster P (1989). The molecular Quasi-species. Adv Chem Phys.

[B15] Goldstein R, Luthey-Schulten, Wolynes P (1992). Optimal Protein-Folding Codes from Spin-Glass Theory. Proc Nat Acad Sci USA.

[B16] Wagner A (2002). Selection after gene duplication: a view from the genome. Genome Biology.

[B17] Lynch M, O'Hely M, Walsh B, Force A (2001). The Probability of Preservation of a Newly Arisen Gene Duplicate. Genetics.

[B18] Ludwig M, Kreitman M (1995). Evolutionary Dynamics of the Enhancer region of even-skipped in Drosophila. Mol Biol Evol.

[B19] Ludwig M, Patel N, Kreitman M (1998). Functional analysis of eve stripe 2 enhancer evolution in Drosophila: rules governing conservation and change. Development.

[B20] Dermitzakis E, Bergman C, Clark A (2002). Tracing the evolutionary history of Drosophila regulatory regions with models that identify transcription factor binding sites. Mol Biol Evol.

[B21] Scemama J, Hunter M, McCallum J, Prince V, Stellwag E (2002). Evolutionary divergence of vertebrate Hoxb2 expression patterns and transcriptional regulatory loci. J Exp Zool.

[B22] Arnosti DN (2003). Analysis and function of transcriptional regulatory elements: Insights from Drosophila. Ann Review Entymology.

[B23] Costas J, Casares F, Vieira J (2003). Turnover of binding sites for transcription factors involved in early Drosophila development. Gene.

[B24] McGregor A, Shaw P, Hancock J, Bopp D, Hediger M, Wratten N, Dover G (2001). Rapid restructuring of bicoid-dependent hunchback promoters within and between Dipteran species: Implications for molecular evolution. Evolution and Development.

[B25] Shapiro J (1999). Transposable elements as the key to a 21st century view of evolution. Genetica.

[B26] Wray G, Hahn M, Abouheif E, Balhoff J, Pizer M, Rockman M, Romano L (2003). The evolution of transcriptional regulation in eukaryotes. Mol Biol Evol.

[B27] MacArthur S, Brookfield J (2004). Expected rates and modes of evolution of enhancer sequences. Mol Biol Evol.

[B28] Fields D, He Y, Al-Uzri A, Stormo G (1997). Quantitative specificity of the mnt repression. J Mol Biol.

[B29] Oda M, Furukawa K, Ogata K, Sarai A, Nakamura H (1998). Thermodynamics of specific and non-specific DNA binding by the c-Myb DNA-binding domain. J Mol Biol.

[B30] Sarai A, Takeda Y (1989). RT Lambda repressor recognizes the approximately 2-fold symmetric half-operator sequences asymmetrically. Proc Nat Acad Sci USA.

[B31] van Opijnen T, Jeeninga R, Boerlijst M, Pollakis G, Zetterberg V, Salminen M, Berkhout B (2004). Human immunodeficiency virus type 1 subtypes have a distinct long terminal repeat that determines the replication rate in a host-cell-specific manner. J Virol.

[B32] Peliti L (2002). Quasispecies evolution in general mean-field landscapes. Europhys Lett.

[B33] Schlötterer C, Hauser MT, v Haeseler A, Tautz D (1994). Comparative evolutionary analysis of rDNA ITS regions in Drosophila. Mol Biol Evol.

[B34] Kimura M, Ohta T (1969). The average number of generations until fixation of a mutant gene in a finite population. Genetics.

[B35] Kingman J (1978). A simple model for the balance between selection and mutation. J Appl Prob.

[B36] Ohta, Tachida (1990). Theoretical Study of Near Neutrality. I. Heterozygosity and Rate of Mutant Substitution. Genetics.

[B37] Kimura M (1962). On the probability of fixation of mutant genes in a population. Genetics.

[B38] Begun D, Aquadro C (1992). Levels of naturally occuring DNA polymorphism correlate with recombination rates in D. melanogaster. Nature.

[B39] Müller-Hill B (1996). The lac operon.

[B40] Hahn M, Stajich J, Wray G (2003). The effects of selection against spurious transcription factor binding sites. Mol Biol Evol.

[B41] Jenkins D, Ortori C, Brookfield J (1995). A test for adaptive change in DNA sequences controlling transcription. Proc R Soc Lond.

[B42] Moses A, Chang D, Kellis M, Lander E, Eisen M (2003). Position specific variation in the rate of evolution in transcription factor binding sites. BMC Evolutionary Biology.

[B43] Ludwig M, Bergman C, Patel N, Kreitman M (2000). Evidence for stabilizing selection in a eukaryotic enhancer element. Nature.

